# Influence of ethnicity on the distribution of genetic polymorphisms associated with risk of chronic liver disease in South American populations

**DOI:** 10.1186/s12863-015-0255-3

**Published:** 2015-07-29

**Authors:** Ana Cecilia Pontoriero, Julieta Trinks, María Laura Hulaniuk, Mariela Caputo, Lisandro Fortuny, Leandro Burgos Pratx, Analía Frías, Oscar Torres, Félix Nuñez, Adrián Gadano, Pablo Argibay, Daniel Corach, Diego Flichman

**Affiliations:** Instituto de Ciencias Básicas y Medicina Experimental (ICBME), Hospital Italiano de Buenos Aires, Potosí 4240, C1199ACL Buenos Aires, Argentina; National Scientific and Technical Research Council (CONICET), Av. Rivadavia 1917, C1033AAJ Buenos Aires, Argentina; Servicio de Huellas Digitales Genéticas, Facultad de Farmacia y Bioquímica, Universidad de Buenos Aires, Junín 954, C1113AAD Buenos Aires, Argentina; Servicio de Medicina Transfusional, Hospital Italiano de Buenos Aires, Juan D. Perón 4190, C1181ACH Buenos Aires, Argentina; Servicio de Medicina Transfusional, Hospital Materno Infantil “Ramón Sardá”, Esteban de Luca 2151, C1246ABQ Buenos Aires, Argentina; Servicio de Hepatología, Hospital Italiano de Buenos Aires, Juan D. Perón 4190, C1181ACH Buenos Aires, Argentina; Cátedra de Virología, Facultad de Farmacia y Bioquímica, Universidad de Buenos Aires, Junín 954, C1113AAD Buenos Aires, Argentina

**Keywords:** Chronic liver disease, Liver cancer, PNPLA3 gene, VDR gene, Polymorphism, Ethnicity, South America

## Abstract

**Background:**

The global burden of chronic liver disease is rising. Besides environmental, behavioral, viral and metabolic factors, genetic polymorphisms in patatin-like phospholipase-3 (*PNPLA3)* and vitamin D receptor (*VDR*) genes have been related to the development of chronic liver disease and progression towards liver cancer. Although their prevalence differs remarkably among ethnic groups, the frequency of these polymorphisms in South American populations -whose genetic background is highly admixed- has been poorly studied. Hence, the aim of this study was to characterize polymorphisms related to chronic liver disease and their association with the genetic ancestry of South American populations.

**Results:**

DNA samples from 258 healthy unrelated male volunteers were analyzed. The frequencies of G and C alleles of rs738409 polymorphism (*PNPLA3* gene) were 74 % and 26 %, respectively; whereas the bAt (CCA) haplotype (*VDR* gene) was observed in 32.5 % of the samples. The GG genotype of *PNPLA3* rs738409 and the bAt (CCA) haplotype -associated with an increased risk of chronic liver disease and progression towards liver cancer- were significantly more frequent among samples exhibiting maternal and paternal Native American haplogroups (63.7 % and 64.6 %), intermediate among admixed samples (45.1 % and 44.9 %; *p* = 0.03) and the lowest for Non-native American ancestry (30.1 % and 29.6 %; *p* = 0.001 and *p* = 0.0008).

**Conclusions:**

These results suggest that individuals with Native American ancestry might have a high risk of chronic liver disorders and cancer. Furthermore, these data not only support the molecular evaluation of ancestry in multi-ethnic population studies, but also suggest that the characterization of these variants in South American populations may be useful for establishing public health policies aimed at high risk ethnic communities.

**Electronic supplementary material:**

The online version of this article (doi:10.1186/s12863-015-0255-3) contains supplementary material, which is available to authorized users.

## Background

The global health and economic burden of chronic liver disease is substantial and on the rise, due in part to the growing incidence of hepatic steatosis and cirrhosis [1, 2]. The increase in cirrhosis, along with a surge in the prevalence of its risk factors such as alcohol abuse and infection with hepatitis B virus (HBV) and/or hepatitis C virus (HCV), has resulted in a growing incidence of primary liver cancer [3]. The major histological subtype of primary liver malignancies, hepatocellular carcinoma (HCC), represents the end stage of the natural history of chronic liver disease and has become the third leading cause of cancer-related mortality worldwide and the major indication for liver transplant in Europe and USA [4].

Hepatic steatosis is the most common pathological finding in liver biopsies around the world [5]. Although its association with liver inflammation is an evidence suggestive of alcohol-induced liver injury, it has also been described in individuals with no significant history of alcohol ingestion [6, 7]. The term non-alcoholic fatty liver disease (NAFLD) has been adopted to cover the full spectrum of metabolic fatty liver disorders, as it ranges from simple steatosis to non-alcoholic steatohepatitis (NASH) and fibrosis with severe disruption of the normal architecture and function of the liver leading to cirrhosis [6, 7]. Increasing rates of NAFLD and NASH -the most common forms of chronic liver disease in Western countries- have been reported as the worldwide prevalence of obesity continues to rise [5–7]. In turn, obesity, NAFLD and NASH are increasingly recognized triggers involved in the development of both cirrhosis and HCC [8].

There is consistent evidence that the burden of chronic liver disease is greater among certain ethnic groups, particularly in areas characterized by high rates of chronic infection with hepatitis viruses [9]. Moreover, racial disparities in the prevalence and clinical presentation of chronic liver disorders have frequently been reported. In fact, Hispanics have a higher prevalence of steatohepatitis, NAFLD and cirrhosis, whereas African-Americans are less prone to develop liver failure [10–12].

Besides viral, environmental, behavioral, and metabolic features, genetic factors have also been implicated in the different susceptibility to develop HCC [13]. In the last few years, several studies have established the contribution of single nucleotide polymorphisms (SNPs) in different genes -such as the patatin-like phospholipase-3 (*PNPLA3*), vitamin D receptor (*VDR*), transmembrane 6 superfamily member 2 (*TM6SF2*), beta-parvin (*PARVB*) and sorting and assembly machinery component 50 (*SAMM50*) genes- as causal variants for chronic liver diseases and their progression towards HCC [14–17]. This study will focus only on the *PNPLA3* and *VDR* polymorphisms.

A genome-wide association study in a population comprising Hispanic, African-American and Caucasian subjects revealed that the SNP rs738409C > G, which encodes an isoleucine-to-methionine substitution at position 148 (I148M) in the *PNPLA3* gene, is the strongest determinant of hepatic steatosis [18]. Many other studies in Asian, European and Latin American populations have since confirmed this association and shown the implication of this genetic variant in chronic liver disorders ranging from NAFLD and NASH to cirrhosis and HCC [19–22].

Moreover, the progression of chronic liver diseases is related to the presence of genetic polymorphisms in the *VDR* gene. In fact, decreased vitamin D levels have increasingly been recognized in various forms of chronic liver disease and associated with advanced fibrosis [23, 24]. Patients with NAFLD were found to present with reduced vitamin D levels, which were closely related to the histological severity of hepatic steatosis, necroinflammation and fibrosis [25, 26].

Several polymorphic sites -such as the bAt (CCA) haplotype consisting of the combination of SNPs rs1544410C (formerly BsmI), rs7975232C (ApaI) and rs731236A (TaqI)- have been described in the *VDR* gene [27]. These combined variants showed a significant association with the fibrosis progression rate and the occurrence of cirrhosis and HCC in Caucasian and Asian patients diagnosed with alcohol-induced liver injury as well as in those chronically infected with HCV [28–30].

The prevalence of these polymorphisms differs among ethnic groups [18, 27]. However, there is a paucity of information about South American populations, whose genetic background is highly admixed as a result of generations of intermixing between various groups, including Native Americans, Spanish conquistadores, Africans and a large European immigrant population that arrived between 1870 and 1950. In the Argentinean population, for example, further sources of admixture have been introduced by local migration from the rural areas to the cities between 1930 and 1980, as well as by immigration from other South American countries [31, 32]. Hence, the aim of this study was to determine the prevalence of these genetic polymorphisms and their association with the genetic ancestry of South American populations, which may provide useful knowledge for establishing public health policies aimed at high risk ethnic populations.

## Results

The SNPs in *PNPLA3* and *VDR* genes, associated to the development of chronic liver disease and progression towards liver cancer, have been characterized in 258 healthy unrelated male volunteers. The allele and genotype frequencies of these SNPs are presented in Table 1. No significant differences in age were observed among the individuals carrying the different alleles. The distribution of homozygous and heterozygous carriers was consistent with the expectations of the Hardy-Weinberg equilibrium (chi-square goodness-of-fit test: *p* > 0.05 for all SNPs in all groups).

**Table 1:** Allele and genotype frequencies of SNPs rs738409 (*PNPLA3*), rs1544410, rs7975232, rs731236 and bAt (CCA) haplotype (*VDR*)

	Allele frequency		Genotype frequency (%)	
*PNPLA3*				
rs738409	G:	0.74	GG:	55.4
	C:	0.26	GC/CC:	44.6
*VDR*				
rs1544410 (BsmI)	C:	0.78	CC:	58.9
	T:	0.22	CT/TT:	41.1
rs7975232 (ApaI)	C:	0.70	CC:	50.4
	A:	0.30	CA/AA:	49.6
rs731236 (TaqI)	A:	0.75	AA:	55.8
	G:	0.25	AG/GG:	44.2
Haplotype			bAt (CCA):	32.5
			Others haplotypes:	67.5

**Fig. 1:**
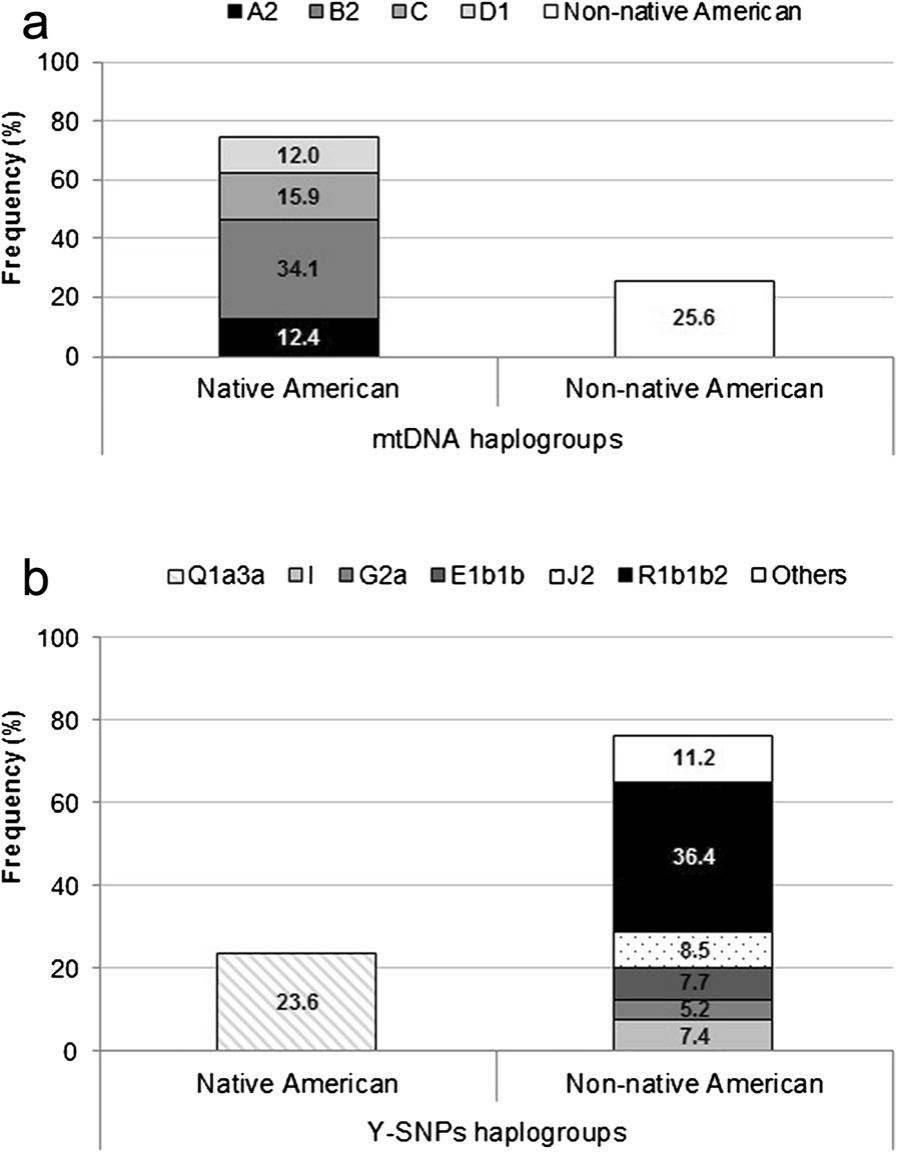
Genetic ancestry of the study population. Prevalence of (**a**) mitochondrial DNA (mtDNA) haplogroups and (**b**) Y-chromosome single-nucleotide polymorphisms (Y-SNPs) among the studied population

The molecular evaluation of maternal ancestry reveals that 74.4 % of the 258 individuals exhibited Native American lineages (haplogroups A2, B2, C and D1; Fig. 1a); whereas, as regards paternal ancestry, only 23.6 % of the samples showed the Native American Q1a3a haplogroup (Fig. 1b). Moreover, the combined analysis of mitochondrial DNA (mtDNA) and Y-chromosome single-nucleotide polymorphisms (Y-SNPs) in each of the 258 recruited individuals revealed that more than half of them (57.8 %) exhibited maternal Native American haplogroups and paternal Non-native American ancestry (admixed samples); whereas 17.8 % and 24.4 % of the studied samples showed Native American and Non-native American ancestry, respectively.

**Fig. 2:**
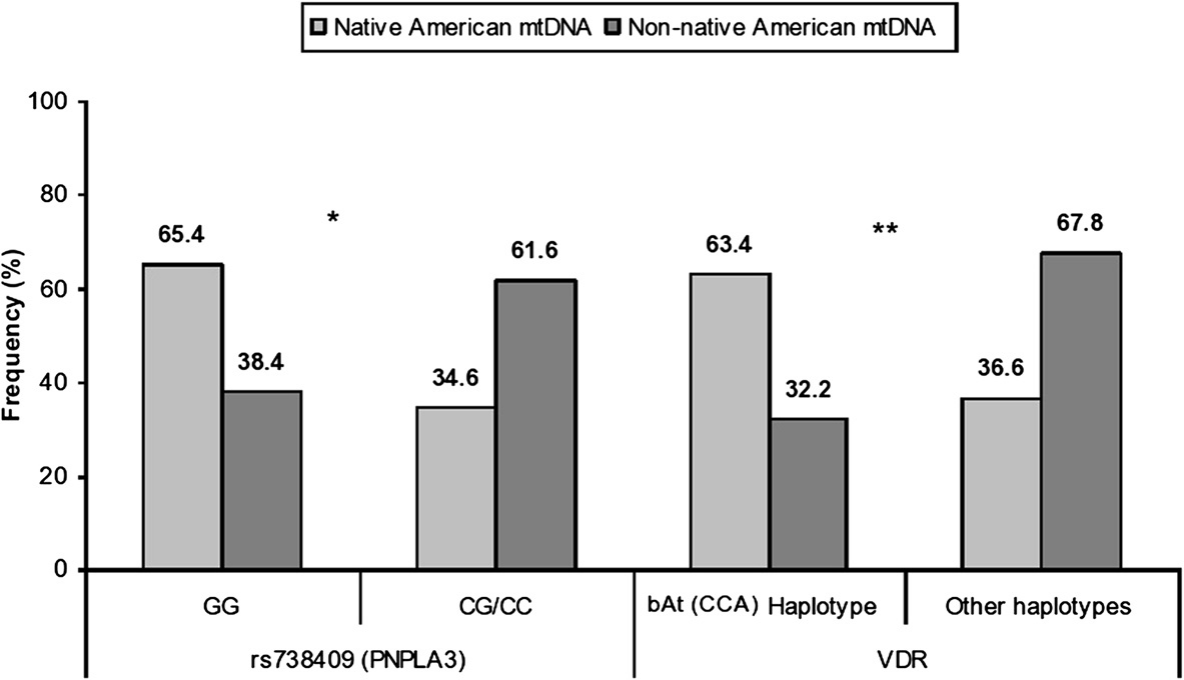
Influence of maternal ethnic components on the genotype distribution. Prevalence of SNP rs738409 (*PNPLA3* gene) and bAt (CCA) haplotype (*VDR* gene) among samples of Native American and Non-native American maternal ancestry (*p = 0.0008, **p = 0.0005)

**Fig. 3:**
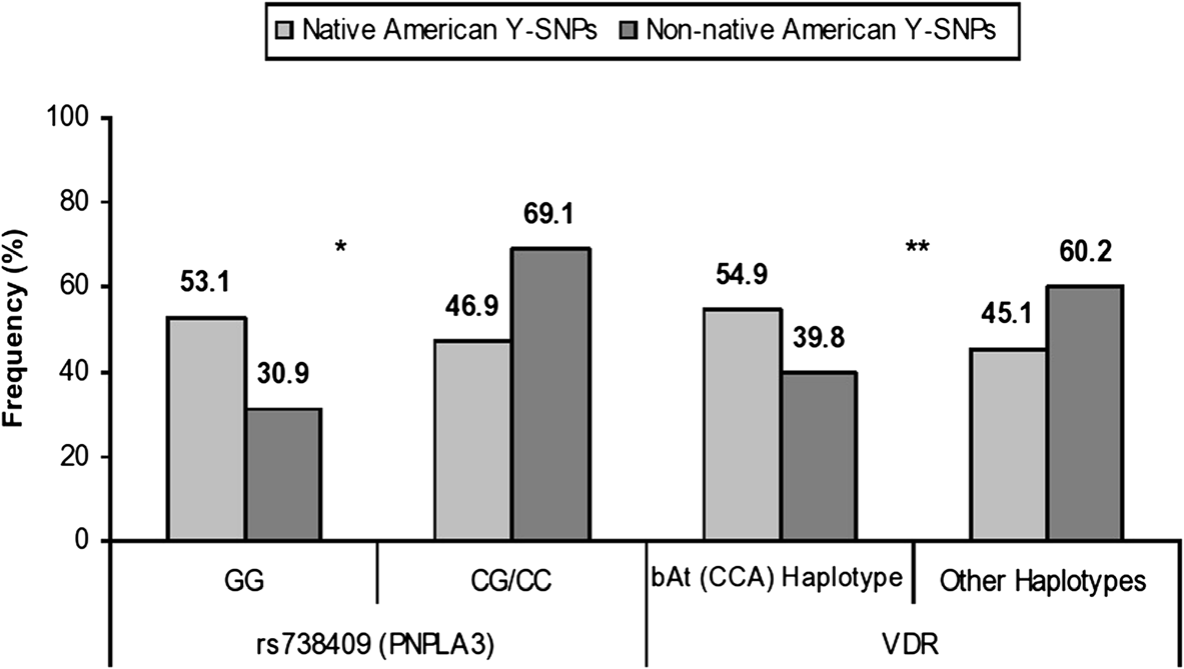
Influence of paternal ethnic components on the genotype distribution. Prevalence of SNP rs738409 (*PNPLA3* gene) and bAt (CCA) haplotype (*VDR* gene) among samples of Native American and Non-native American paternal ancestry (*p = 0.0388, **p = 0.0424)

The four studied SNPs were unevenly distributed among individuals with Non-native American ancestry compared with those with Native American ancestry (maternal or paternal lineages). In fact, the frequencies of bAt (CCA) haplotype (*VDR* gene) and the GG genotype of rs738409 polymorphism (*PNPLA3* gene) were significantly higher among samples with Native American maternal ancestry (63.4 % and 65.4 %, respectively) compared with those with Non-native American maternal haplogroups (32.2 % and 38.4 %, respectively) (*p* = 0.0005 and *p* = 0.0008, respectively; Fig. 2). In addition, these frequencies were unevenly distributed among individuals with Native American paternal ancestry compared with those with Non-native American paternal ancestry, being significantly higher in those with Native American haplogroups (54.9 % and 53.1 %, respectively) compared with those with Non-native American haplogroups (39.8 % and 30.9 %, respectively) (*p* = 0.0424 and *p* = 0.0388, respectively; Fig. 3). Similar results were observed when examining the relationship between polymorphisms in *VDR* gene and ancestry (Additional files 1 and 2).

**Fig. 4:**
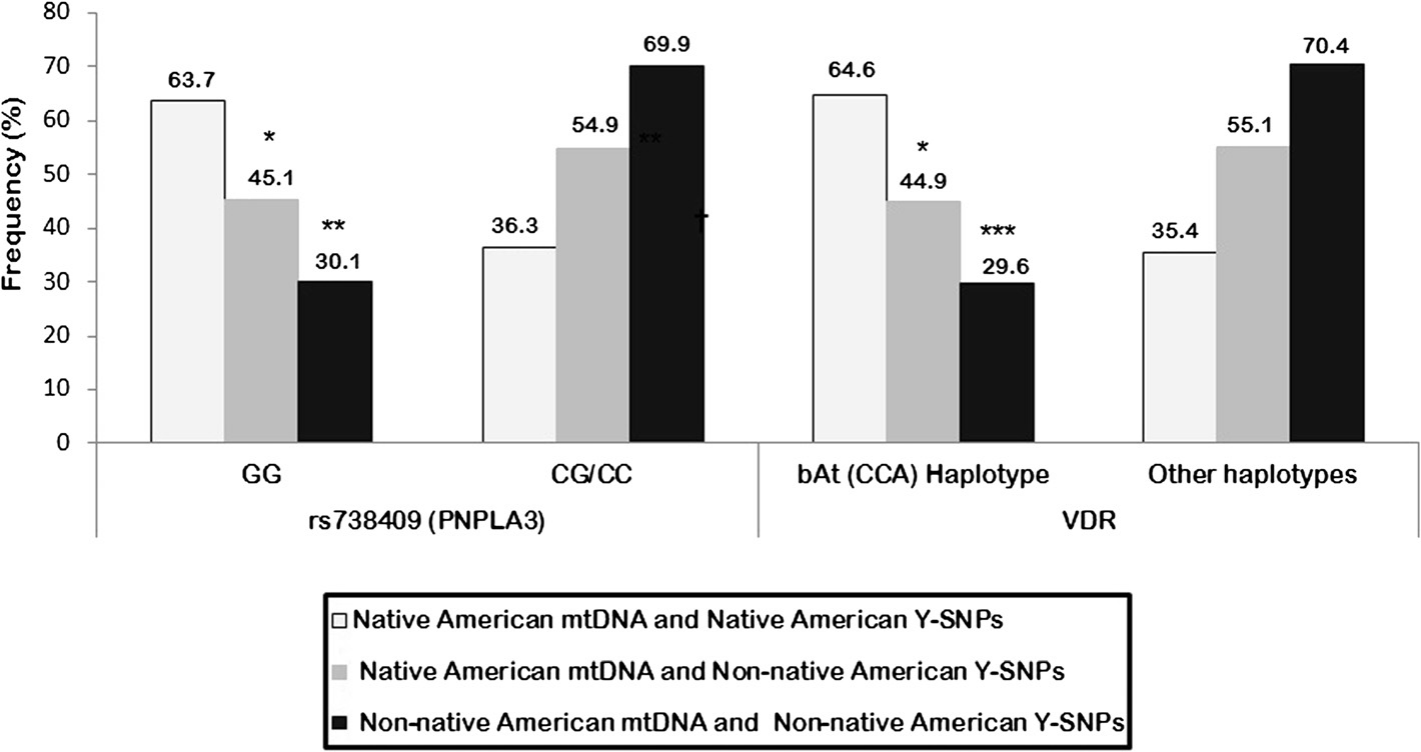
Influence of maternal and paternal ethnic components on the genotype distribution. Prevalence of SNP rs738409 (*PNPLA3* gene) and bAt (CCA) haplotype (*VDR* gene) among samples of maternal and paternal Native American ancestry, Native American maternal ancestry and Non-native American paternal ancestry (admixed samples) and Non-native American maternal and paternal ancestry. *p = 0.03 when comparing samples with maternal and paternal Native American ancestry with the admixed group; **p = 0.001 and ***p = 0.0008 when comparing samples with maternal and paternal Native American ancestry with those exhibiting Non-native American maternal and paternal ancestry

When the prevalence of the SNP rs738409 (*PNPLA3*) was determined according to the combined ancestry of the samples (maternal and paternal lineages), the unfavorable genotype GG was significantly higher among samples with Native American lineages (63.7 %), intermediate among admixed samples (45.1 %, *p* = 0.03) and the lowest for samples with Non-native American ancestry (30.1 %, *p* = 0.001; Fig. 4).

Similar results were found when the three polymorphisms in *VDR* gene were examined (Additional file 3). As a consequence, the frequency of the bAt (CCA) haplotype -which is a result of the combination of the three SNPs- was significantly higher among samples with maternal and paternal Native American haplogroups (64.6 %), intermediate among admixed samples (44.9 %, *p* = 0.03) and the lowest for samples with Non-native American ancestry (29.6 %, *p* = 0.0008; Fig. 4).

## Discussion

Chronic liver disease is an important cause of morbidity and mortality around the world. The ethnic disparities reported in its burden, prevalence and clinical presentation are likely attributed to the interaction between environmental, behavioral, and genetic factors [1, 9]. To the best of our knowledge, this study is the first to analyze ethnicity and polymorphisms associated with susceptibility to chronic liver disease and progression towards HCC in South American populations.

The results presented herein reveal that the distribution of polymorphisms related to the development of chronic liver disease and progression towards liver cancer exhibits statistical significant differences according to ethnicity. In fact, the prevalence of host genetic factors associated with risk of liver steatosis, fibrosis and cirrhosis is significantly lower in individuals with Non-native American ancestry.

In addition to ethnicity, other risk factors have been associated with the development of chronic liver disease, such as older age, male gender, alcohol abuse and HBV and/or HCV infection [1]. To ensure a representative sample of the general population in the region, 258 unrelated male volunteers were enrolled by two blood banks in Buenos Aires city. The exclusion of HBV and/or HCV infected subjects did not affect sample’s representativeness as prevalence of HBV and HCV infection in Argentina is lower than 1 % [33].

Moreover, the proportion of each ancestry pattern in the collected sample was consistent with previously reported frequencies in the general population [32], and therefore, no ethnic group was over or underrepresented in the sample. In this area of Argentina, the more prevalent ancestry components for maternal and paternal lineages are the European and the Native American, followed -to a lesser extent- by African haplogroups [32]. Therefore, these data suggest that the samples with Non-native American maternal and/or paternal haplogroups analyzed in this study would predominantly exhibit the European component. In addition, the process of admixture was -as in the rest of South America- sex-biased, generally involving a Native American maternal lineage and a Non-native American paternal lineage [32]. This finding is consistent with fact that the admixed group included in this study was characterized by Native American maternal ancestry and Non-native American paternal haplogroups.

In general, men are 2-fold more likely to die from chronic liver disease and cirrhosis than are women. Liver transplant occurs less commonly in women than in men, with variable disease outcomes based on etiology [34]. A prospective study which analyzed epidemiological aspects of HCC in South America revealed that the situation in this region is no different from that in the rest of the world [35]. In this study, only male subjects were enrolled to assess both maternal and paternal lineages and determine the frequency of admixture in the studied population. In spite of this, the genotype prevalence of the studied SNPs must be considered as representative of the general population as it could not be modified by the lack of women in the recruited sample. However, it must be acknowledged that the interpretation of the results presented in this study may be affected by the possibility of sex difference in the genetic association of these polymorphisms and disease susceptibility. In fact, a negative correlation between male sex and the effect of rs738409 on liver fat content has been reported in NAFLD patients [36].

In this report, the prevalence of the genetic polymorphisms associated with risk of chronic liver disease in Non-native and Native American samples is in agreement with previously described frequencies in European and South American populations [18, 20, 37].

Regarding the GG genotype of SNP rs738409 in *PNPLA3* gene, its frequency was significantly higher in individuals with Native American ancestry when compared with those exhibiting Non-native American haplogroups. In consistency with our results, a high prevalence of the unfavorable genotype was reported in Hispanics and in Argentinean patients with NALFD [18, 20]; but unfortunately, the ancestry components of these previously studied populations remain unknown.

Furthermore, the analysis of *VDR* polymorphisms carried out in our study revealed intermediate frequencies between those reported for European and Asian samples, but similar to those previously published for Hispanics [37]. This result could be related to the high admixture levels observed in these populations, which could be explained by the combined ancestry of mestizos and the fact that Native American populations are descendants of nomadic Asian communities [37].

In different populations, haplotype structure analysis of *VDR* gene has shown high genetic heterogeneity involving different haplotype blocks [37, 38]. In fact, studies carried out in admixed populations has revealed the importance of genetic heterogeneity since linkage disequilibrium increases or breaks down differently in different populations [39, 40]. Considering the recent admixture of Latin American people, the variation in the patterns of linkage disequilibrium is not surprising in view of demographic events and genetic factors such as drift and recombination during the process of admixture.

From the genetic perspective, Hispanics or Latinos generally represent an intricate mixture of European, Native American, and African lineages, with the proportion of each ancestry component typically depending on country or area of origin [41]. Recent studies that have examined the relationships between self-identified ethnicity, self-estimated admixture proportions and genetic marker estimated admixture proportions revealed that Hispanics underestimate their degree of Native American admixture, confirming that ethnic self-identification is likely to be complex due to the heterogeneity in individual admixture proportions and social environments within this group [32, 42]. Due to its complex diversity, data obtained from relatively well-defined ethnic groups cannot be extrapolated to the majority of the South American population.

Interethnic admixture is either common or rapidly increasing in many -if not most- populations as a result of human migrations. Therefore, in agreement with our results, molecular evaluation of ancestry is strongly recommended in studies including this type of multi-ethnic populations [43]. From this point of view, the South American population, with Native American, European and African ancestral roots, and five centuries of vast interethnic admixing, offers a unique model for examining the impact of admixture on the conceptual development and clinical implementation of the results obtained from genomics medicine studies.

The results presented in this study intended to shed some light on the influence of ethnicity on the distribution of genetic polymorphisms associated with chronic liver disease in multi-ethnic and admixed populations. However, due to the multifactorial aspect of chronic liver diseases, the high prevalence of *PNPLA3* and *VDR* genetic factors in Native Americans cannot be considered as the sole reason for the higher susceptibility of this ethnic group. Therefore, for the proper interpretation of the results presented herein, it must be taken into consideration that other genetic (e.g., *SAMM50, PARVB, TM6SF2*), environmental and behavioral factors may -as a whole- contribute to the higher risk of chronic liver disease in Native Americans [1–6, 16, 17]. In fact, the Hispanic population has high rates of obesity, diabetes and metabolic syndrome [44] which suggest that other nutritional and genetic factors exert a role in the increased risk for chronic liver disease in this population, as well. Unfortunately, the prevalence of chronic liver disease in subjects with Native American ethnic components in the studied area remains unknown to clarify this hypothesis.

In future decades, the prevalence of risk factors for chronic liver diseases, such as obesity, alcohol abuse and HCV chronic infection, is expected to rise [1, 2]. Hence, the identification of effective genetic markers for chronic liver disorders would be priceless as it would allow us to stratify populations according to susceptibility [45]. As a consequence, in areas exhibiting a highly admixed genetic background with a significant contribution of Native American ancestry components -and, therefore, less favorable genetic variants-, the results presented in this study are of substantial relevance for the regional public health.

## Conclusion

In conclusion, this study shows -for the first time- that more than 60 % of individuals with Native American ancestry are carriers of high risk genetic polymorphisms related to chronic liver disease and progression towards liver cancer. These data not only support the molecular evaluation of ancestry in multi-ethnic population studies, but also suggest that the characterization of these variants among the healthy South American population may be useful for establishing public health policies aimed at the organization of awareness-raising campaigns for high risk ethnic communities.

## Methods

### Study population

During the period 2012–2013, a total of 258 unrelated male volunteers (mean age ± standard deviation: 35.2 years ± 11.5) were recruited by the DNA and blood bank at the Italian Hospital of Buenos Aires and the blood bank at the “Sardá” Maternity Hospital in Buenos Aires, Argentina. Both blood banks are located in different points of Buenos Aires city and provide attention to all strata of the general population (both Argentinean residents and South American immigrants), reinforcing sample’s representativeness of the population of this region.

Male gender was considered as an inclusion criteria for participation in the study in order to determine both maternal (mitochondrial) and paternal (Y-chromosome) ancestry in all collected samples. Moreover, all enrolled subjects exhibited negative serology results for HBV and HCV infection (AxSYM, Abbott, Chicago, IL, USA) to avoid the dominant role that those viruses play on the development of chronic liver disease.

### Ethics, consent and permissions

This study has been performed in accordance with the Declaration of Helsinki. Informed consent was obtained from each subject according to the experimental research protocol, approved by the Ethics Committee on Research from the Italian Hospital of Buenos Aires (CEPI N° 1918).

### Isolation of genomic DNA and genotype of PNPLA3 and VDR

Genomic DNA was extracted from 1 ml of EDTA blood by using FlexiGene® DNA Kit (QIAGEN, GmbH, Hilden, Germany) following the manufacturer’s protocol.

Primers for SNPs rs738409 (*PNPLA3*) were designed by using Primer3 software [46]. A 668-bp fragment of *PNPLA3* gene was amplified by the polymerase chain reaction (PCR) technique using primers 5′-CGA TCT AGC CCC TTT CAG TC-3′ (forward) and 5′-GCA GAT TAA GTG AAC CAG CC-3′ (reverse). PCR reaction was carried out for 30 cycles consisting of denaturation for 30 s at 94 °C, annealing for 30 s at 62 °C and extension for 1 min at 72 °C. With regard to SNPs for *VDR* gene, the sets of primers used for amplification and their PCR cycling conditions were obtained from previously published studies [47, 48].

The PCR amplified fragments were bi-directionally sequenced using Big-Dye Termination chemistry system (Applied Biosystems, Life Technologies Corp., Foster City, CA, USA). In order to discriminate between homozygotes and heterozygotes, the sequencing chromatogram was examined by using BioEdit Sequence Alignment Editor version 7.1.3.0.

### Molecular evaluation of ancestry

Haplogroups in mtDNA (haplogroups A2, B2, C and D1) and Y-SNPs (haplogroups E1b1b, G2a, I, J2, R1b1b2, Q1a3a) were assessed by Real-Time PCR followed by High Resolution Melting as previously described [49].

### Statistical analyses

Statistical analyses were carried out using Fisher’s exact test (group by group comparisons) and a value *p* < 0.05 was considered as statistically significant. Furthermore, Holm-Bonferroni correction was used to address the problem of multiple hypothesis testing.

The correlation between the observed number of homozygous and heterozygous individuals and the numbers statistically expected from the Hardy-Weinberg equilibrium was assessed by the Chi-square goodness-of-fit test.

## Additional files

Additional file 1:
**Prevalence of SNPs rs1544410 (BsmI), rs7975232 (ApaI) and rs731236 (TaqI) of** ***VDR*** **gene among samples with Native American maternal ancestry and Non-native American maternal ancestry.** **p* = 0.0008, ***p* = 0.0172, ****p* = 0.0033. (JPEG 80 kb) Additional file 2:
**Prevalence of SNPs rs1544410 (BsmI), rs7975232 (ApaI) and rs731236 (TaqI) of** ***VDR*** **gene among samples with Native American paternal ancestry and Non-native American paternal ancestry.** **p* = 0.0304, ***p* = 0.0588, ****p* = 0.004. (JPEG 87 kb) Additional file 3:
**Prevalence of SNPs rs1544410 (BsmI), rs7975232 (ApaI) and rs731236 (TaqI) of the** ***VDR*** **gene among samples of maternal and paternal Native American ancestry, Native American maternal ancestry and Non-native American paternal ancestry (admixed samples) and Non-native American maternal and paternal ancestry.** **p* = 0.009, ***p* = 0.0261 and ****p* = 0.0396 when comparing samples with maternal and paternal Native American ancestry with the admixed group; †*p* = 0.0005 and ‡*p* = 0.001 when comparing samples with maternal and paternal Native American ancestry with those exhibiting Non-native American maternal and paternal ancestry. (JPEG 86 kb)

## Abbreviations

EDTA: Ethylenediaminetetraacetic acid
HBV: Hepatitis B virus
HCC: Hepatocellular carcinoma
HCV: Hepatitis C virus
mtDNA: Mitochondrial DNA
n: Sample size
NAFLD: Non-alcoholic fatty liver disease
NASH: Non-alcoholic steatohepatitis
PCR: Polymerase chain reaction
PNPLA3: Patatin-like phospholipase 3
SD: Standard deviation
SNP: Single-nucleotide polymorphism
VDR: Vitamin D receptor
Y-SNPs: Y-chromosome single-nucleotide polymorphisms
