# Supplementary figures and images for: Influence of ethnicity on the distribution of genetic polymorphisms associated with risk of chronic liver disease in South American populations
Source: BMC Genet. 2015 Jul 29;16:93. doi: 10.1186/s12863-015-0255-3 (PMC4518515; doi:10.1186/s12863-015-0255-3)

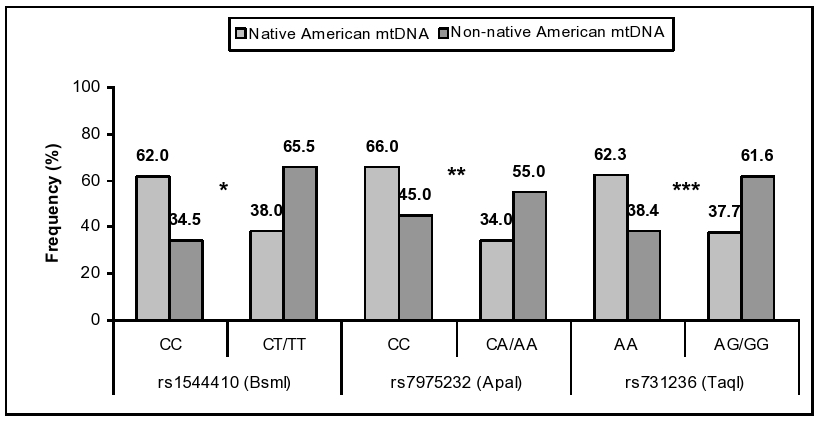

Supplement: Additional file 1: — Prevalence of SNPs rs1544410 (BsmI), rs7975232 (ApaI) and rs731236 (TaqI) of VDR gene among samples with Native American maternal ancestry and Non-native American maternal ancestry. *p = 0.0008, **p = 0.0172, ***p = 0.0033. (JPEG 80 kb) [file 12863_2015_255_MOESM1_ESM.jpg]

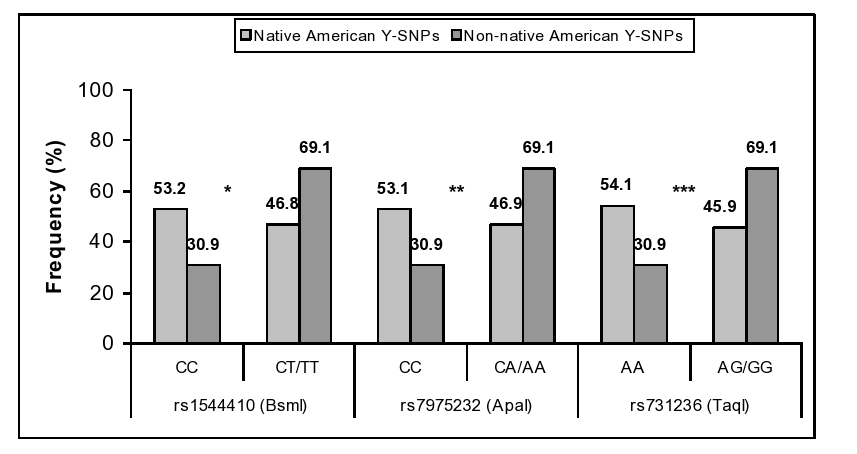

Supplement: Additional file 2: — Prevalence of SNPs rs1544410 (BsmI), rs7975232 (ApaI) and rs731236 (TaqI) of VDR gene among samples with Native American paternal ancestry and Non-native American paternal ancestry. *p = 0.0304, **p = 0.0588, ***p = 0.004. (JPEG 87 kb) [file 12863_2015_255_MOESM2_ESM.jpg]

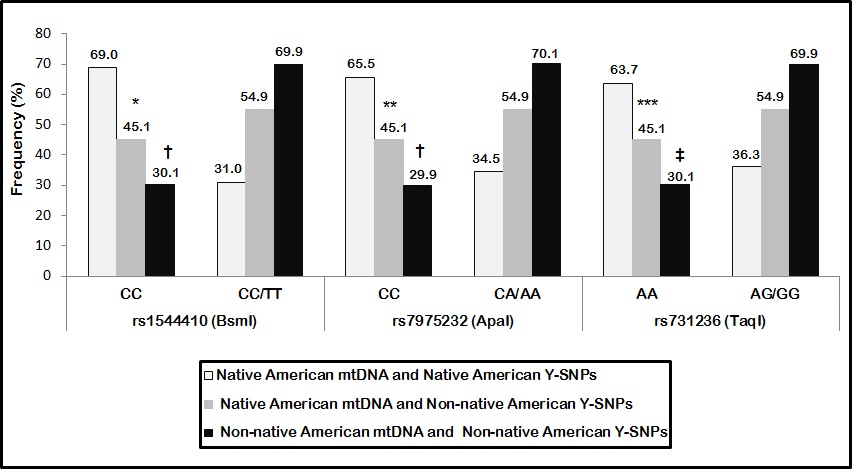

Supplement: Additional file 3: — Prevalence of SNPs rs1544410 (BsmI), rs7975232 (ApaI) and rs731236 (TaqI) of the VDR gene among samples of maternal and paternal Native American ancestry, Native American maternal ancestry and Non-native American paternal ancestry (admixed samples) and Non-native American maternal and paternal ancestry. *p = 0.009, **p = 0.0261 and ***p = 0.0396 when comparing samples with maternal and paternal Native American ancestry with the admixed group; †p = 0.0005 and ‡p = 0.001 when comparing samples with maternal and paternal Native American ancestry with those exhibiting Non-native American maternal and paternal ancestry. (JPEG 86 kb) [file 12863_2015_255_MOESM3_ESM.jpg]
